# Comparison of guideline concordant antibiotic prophylaxis in Veterans Affairs and non-Veterans Affairs dental settings among those with cardiac conditions or prosthetic joints

**DOI:** 10.1186/s12879-023-08400-y

**Published:** 2023-06-23

**Authors:** Swetha Ramanathan, Charlesnika T. Evans, Ronald C. Hershow, Gregory S. Calip, Susan Rowan, Colin Hubbard, Katie J. Suda

**Affiliations:** 1 School of Public Heath, University of Illinois at Chicago, Chicago, IL USA; 2 Center of Innovation for Complex Chronic Healthcare, Hines VA Hospital, Hines, IL USA; 3 Department of Preventive Medicine and Center for Health Services and Outcomes Research, Northwestern University Feinberg School of Medicine, Chicago, IL USA; 4 College of Pharmacy, University of Illinois at Chicago, Chicago, IL USA; 5 College of Dentistry, University of Illinois at Chicago, Chicago, IL USA; 6 Division of Hospital Medicine, Department of Medicine, University of California San Francisco, San Francisco, CA USA; 7 Center for Health Equity Research and Promotion, VA Pittsburgh Healthcare System, 3609 Forbes Ave. Suite 2, Pittsburgh, PA USA; 8 Department of Medicine, University of Pittsburgh, Pittsburgh, PA USA

**Keywords:** Dentists, Antibiotics, Prophylaxis, VA Dentistry, Private Practice, Guideline Concordant Prescribing

## Abstract

**Background:**

No research has been conducted to assess whether antibiotic prophylaxis prescribing differs by dental setting. Therefore, the goal of this study was to compare the prescribing of antibiotic prophylaxis in Veterans Affairs (VA) and non-Veterans Affairs settings.

**Methods:**

This was a retrospective study of veteran and non-veteran dental patients with cardiac conditions or prosthetic joints between 2015–2017. Multivariable log binomial regression analysis was conducted to compare concordant prescribing by setting with a sub-analysis for errors of dosing based on antibiotic duration (i.e., days prescribed).

**Results:**

A total of 61,124 dental visits that received a prophylactic antibiotic were included. Most were male (61.0%), and 55 years of age or older (76.2%). Nearly a third (32.7%) received guideline concordant prophylaxis. VA dental settings had a lower prevalence of guideline concordant prescribing compared to non-VA settings in unadjusted results (unadjusted prevalence ratio [uPR] = 0.92, 95% CI: 0.90–0.95). After adjustment, prevalence of guideline concordant prescribing was higher in those with prosthetic joints in the VA setting (adjusted prevalence ratio [aPR] = 1.73, 95% CI: 1.59–1.88), with no difference identified in those without a prosthetic joint (aPR = 0.99, 95% CI: 0.96–1.01). Concordance of dosing was higher in VA compared to non-VA settings (aPR = 1.11, 95% CI: 1.07–1.15).

**Conclusions:**

VA has a higher prevalence of guideline concordant prescribing among those with prosthetic joints and when assessing dosing errors. Though the presence of an integrated electronic health record (EHR) may be contributing to these differences, other system or prescriber-related factors may be responsible. Future studies should focus on to what extent the integrated EHR may be responsible for increased guideline concordant prescribing in the VA setting.

**Supplementary Information:**

The online version contains supplementary material available at 10.1186/s12879-023-08400-y.

## Introduction

In the United States, greater than 60% of antibiotic prescriptions are associated with the outpatient setting [1]. A particular concern with outpatient antibiotics is that at least 30% of antibiotics prescribed in primary care settings are unnecessary (i.e., no antibiotic was needed) [2]. One study identified that dentists are the top specialty prescriber of antibiotics, accounting for 10% of all outpatient antibiotic prescriptions [3]. Prior to this finding, dentists had been overlooked regarding discipline specific antimicrobial stewardship efforts.

The primary indications dentists prescribe antibiotics for are infection prophylaxis for the prevention of infective endocarditis (IE) and prosthetic joint infections (PJI) [4]. The most recent guidelines for antibiotic prophylaxis for infective endocarditis and prosthetic joint infection were released in 2007 (updated in 2021) and 2013, respectively [5]. Current guidelines from the American Heart Association (AHA) recommend use of antibiotics for IE prevention in patients with specific cardiac conditions (e.g. patients with prosthetic cardiac valves, previous IE) [6]. Guidelines from the American Academy of Orthopedic Surgeons (AAOS) and the American Dental Association (ADA) rarely recommend the use of antibiotics for prevention of PJIs [5, 7].

Previous dental antibiotic prescribing research has focused on trends, regional variations, and prescribing by dental specialists [8–13]. However, one aspect that has not been studied is whether differences between varying dental settings can impact dental antibiotic prescribing. In the US, there are several distinct dental services. The Department of Veterans Affairs is the largest provider of oral health in the US [14]. In addition, dental care also occurs through private practices and is available with dental insurance or patients pay out-of-pocket for oral health care, as well as other government funded health clinics (ex. Public Health Service). Recent work in the VA have identified that dental specialties (endodontics, oral & maxillofacial surgery, orthodontics, dentofacial orthopedics, periodontics, and prosthodontics), lower complexity facilities, rural treatment settings, and Southern region are associated with higher rates of dental antibiotic prescribing [15] while work in the non-VA setting identified higher clinician density, higher median household income, higher proportion female, higher proportion white, proportion greater than 65 years old, middle tertile of poverty for census region, and rurality as factors associated with high prescribing rates [16]. A unique aspect of the VA compared to most private practices is the VA utilizes an integrated electronic health record (EHR), with access to medical and dental data [17]. Few dental practices and systems apart from the VA have an integrated EHR allowing all providers to access medical and dental data [17]. Previous work has found that EHRs have been associated with improving patient safety [18], eliminating gaps in quality of care provided for underserved patients [19], reducing opioid prescriptions [20], and improving antibiotic prescribing [21–25].

Use of an integrated EHR may be beneficial to dentists prescribing antibiotics for prophylaxis, particularly as dentists need information related to cardiac conditions and prosthetic joints to make informed decisions about antibiotic prophylaxis. To assess whether an integrated EHR may be beneficial for concordant antibiotic prophylaxis, it is necessary to compare two systems with and without an integrated EHR. Therefore, the aim of this study was to compare antibiotic prophylaxis by VA and non-VA dental settings, specifically focusing on guideline concordant antibiotic prophylaxis for those with a cardiac condition and/or prosthetic joint.

## Methods

### Study design

This was a retrospective cross-sectional study of VA and non-VA dental settings from January 1, 2015, through December 31, 2017.

### Study population & data sources

**Fig. 1:**
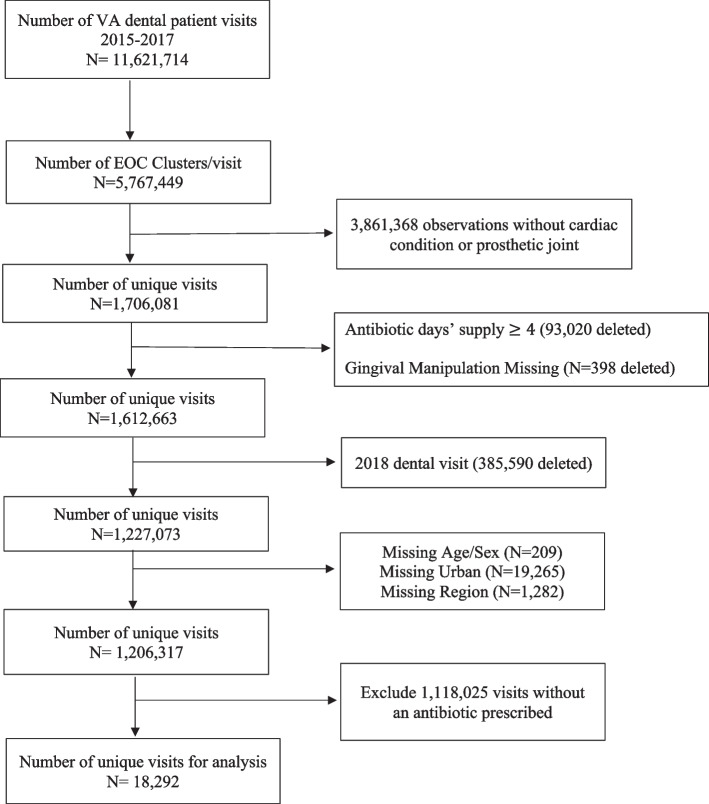
VA Cohort Identification Flowchart

**Fig. 2:**
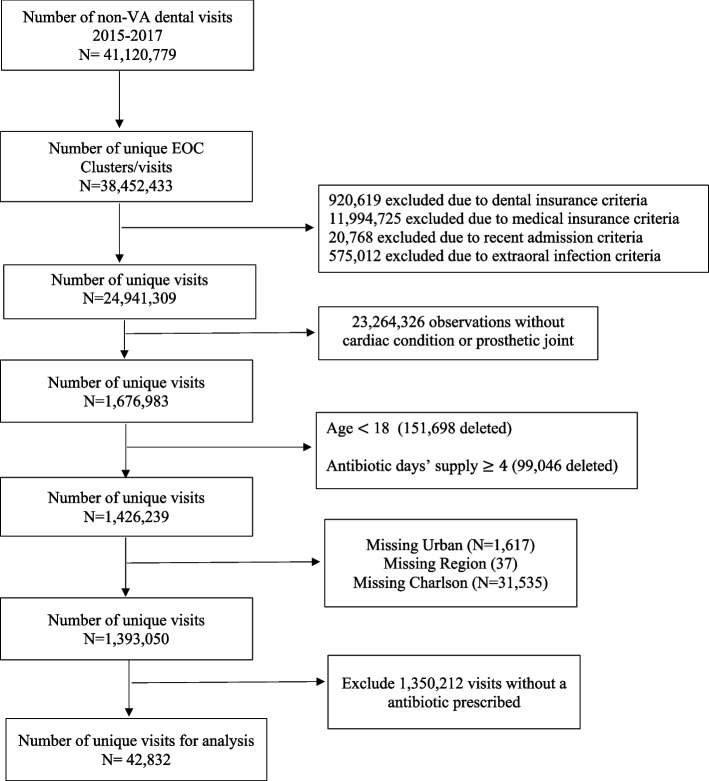
Non- VA Cohort Identification Flowchart

The study population comprised both patients with dental prescriptions and visits from VA and non-VA dental settings. All dental patients were adults 18 years or older with a history of cardiac conditions (e.g., patients with prosthetic cardiac valves, previous IE) or prosthetic joints, identified using International Classification of Diseases (ICD)-9 CM/ ICD-10-CM codes. Data for the VA was obtained from its electronic healthcare data repository, the Corporate Data Warehouse (CDW) while data from Marketscan Commercial Claims/Encounters, Medicare Supplemental, Coordination Benefits and Marketscan Dental Claims was used for non-VA data. The Marketscan data are nationally representative of the insured US population for age, gender, and geographic area, while the dental claims dataset is a convenience sample of 8 million that includes persons with commercial dental insurance or those with Medicare who have opted for commercial dental insurance. The cohort was limited to those that received an antibiotic for a days’ supply of 3 days or less within 7 days prior to the dental visit. Additional exclusion criteria are provided in Figs. 1 and 2.

### Variable definitions

The main independent variable was dental setting, categorized as VA or non-VA. The main outcome variable was guideline concordant prescribing defined as an antibiotic prescribed to a patient with a cardiac condition requiring antibiotic prophylaxis undergoing a dental procedure warranting the antibiotic (i.e., gingival manipulation or perforation of the oral mucosa), according to AHA guidelines, or for a patient with prosthetic joints that did not receive an antibiotic according to the AAOS/ADA guidelines [5, 6, 26]. Procedures that would warrant a prophylaxis include, but are not limited to, tooth extractions, periodontal procedures with bleeding, and oral surgery. An individual may receive multiple antibiotic prescriptions; if at least one antibiotic identified was an appropriate agent (e.g., amoxicillin, cephalexin, azithromycin) according to the AHA guidelines the patient was considered to have a guideline concordant antibiotic.

Additionally, as dental visits are often connected, dental visits within a 7-day window were combined into one episode of care. This was to capture those visits that are related to one another (e.g., a tooth that requires extraction identified at one visit but is extracted at a later visit several days later) and to minimize misclassification of the outcome for connected visits.

Further analyses were conducted to assess errors of prescribing where antibiotics were indicated but dosing was incorrect based on the duration prescribed (e.g., days supply). A secondary outcome measure of guideline concordance was defined as guideline concordant if the individual was prescribed an antibiotic for $\le$ 2 days’ supply while those with a 3 days’ supply was considered guideline discordant.

A number of covariates were assessed in analyses. Age was categorized as: 18–34, 35–44, 45–54, 55–64, and ≥ 65. Gender was assessed as a dichotomous variable (Male/Female). US region was categorized as: Northern, Midwest, Southern, and Western regions. Geographic location was analyzed as a dichotomous variable (Urban/Rural). Dental procedures were identified using the ADA’s Code on Dental Procedures and Nomenclature (CDT Code). CDT codes were grouped into standardized categories of service as defined by the ADA: diagnostic, preventive, restorative, oral and maxillofacial surgery, fixed prosthodontics, adjunctive general services, endodontics, implant services, removable prosthodontics, periodontics, orthodontics, and maxillofacial prosthetics. Each dental procedure was analyzed as a dichotomous variable (Received vs. Did not receive dental procedure) Comorbid conditions were assessed using both the composite Charlson Comorbidity index as well as the individual clinical conditions that are elements of the index, which were identified using ICD-9/10-CM codes [27].

### Statistical analysis

The frequency distribution of concordant prescribing was examined in subgroups defined by patient demographic variables, clinical characteristics, geographic location, and dental procedures using univariate and bivariate statistics. Continuous variables were compared using Student’s T-test while categorical variables were assessed using the Chi-square test of independence. Since the prevalence of the outcome was large, a multivariable log binomial regression analysis was conducted to compare VA and non-VA prescribing [28]. A Poisson regression with robust variance estimation was utilized if the log binomial model did not converge.

Covariates for the multivariable model were selected based on significance (*p* $\le$ 0.05) in bivariate analyses and epidemiological consideration, which included risk factors previously cited in literature. The goal was to construct a parsimonious model that also accounted for factors that have shown to influence prescribing. The final model was constructed using backwards selection, and the Akaike Information Criterion (AIC) was used for the final model selection.

For the sub-analysis assessing guideline concordant dosing based on the duration of the antibiotic prescribed, the main analysis was conducted among those who received a guideline concordant antibiotic according to agent, medical condition, and dental procedure. Both univariate and bivariate analyses were conducted, and multivariable log binomial regression models were used to calculate adjusted prevalence ratios (PR) with 95% confidence intervals. The same covariates and methodology were used for model selection in the sub-analysis, as described previously.

## Results

After all exclusions, 61,124 dental visits received an antibiotic prescription and was the analytic sample identified for this study (Figs. 1 and 2). Of these observations, 18,292 (29.9%) were from the VA setting and 42,832 (70.1%) were from the non-VA setting. The majority were male (61.0%) and 55 years of age or older (76.2%). Over half (62.9%) had a prosthetic joint, 25.7% had a cardiac condition, and 11.4% had both a cardiac condition and prosthetic joint. Most observations were from the Southern region (37.3%) and urban location (84.2%). The average Charlson score was 0.91 (std dev = 1.5, range: 0–17), and the most common comorbidity was diabetes (15%), followed by chronic obstructive pulmonary disease (COPD) (12.4%) and heart failure (7.9%). The most common dental procedures associated with a dental visit were diagnostic (72.2%), preventive (48.6%), and restorative (24.2%) procedures. Guideline concordant antibiotic prophylaxis was observed for 32.7% of visits in the analytic sample. The frequency distribution of other characteristics can be found in Table 1. Comparisons of patient and clinical characteristic by dental setting and distributions of all characteristics by dental setting can be found in the appendix (Supplemental Table 1).

**Table 1:** Distribution of characteristics by Guideline Concordance of Prescribing Decision^a^

		Overall Analysis			Dosing Sub-Analysis		
Variables	Total *N* = 61,124	Concordant (%) *N* = 20,014	Discordant (%) *N* = 41,110	Unadjusted Prevalence Ratio (95% CI)	Concordant (%) *N* = 13,681	Discordant (%) *N* = 6,333	Unadjusted Prevalence Ratio (95% CI)
*Main Independent Variable*							
Dental Setting							
Non-VA	42,832 (70.1)	14,351 (71.7)	28,481 (69.3)	Reference	9.507 (69.5)	4,844 (71.4)	Reference
VA	18,292 (29.9)	5,663 (28.3)	12,629 (30.7)	0.92 (0.90–0.95)	4,174 (30.5)	1,812 (28.6)	1.12 (1.08–1.16)
*Demographics & Clinical Characteristics*							
Mean Age (SD)	59.1 (10.4)	56.8 (13.1)	60.0 (12.0)	1.00 (0.99–1.00)	56.7 (14.0)	56.7 (12.4)	1.00 (0.99–1.00)
Age Group							
18–24	1,799 (2.9)	1,550 (7.7)	249 (0.6)	Reference	1,155 (8.4)	395 (8.4)	Reference
35–44	2,361 (3.9)	1,430 (7.1)	931 (2.3)	0.70 (0.68–0.73)	952 (7.0)	478 (7.6)	0.89 (0.85–0.94)
45–54	10,382 (17.0)	3,752 (18.8)	6,630 (16.1)	0.42 (0.41–0.43)	2,522 (18.4)	1,230 (19.4)	0.90 (0.87–0.94)
55–64	33,641 (55.0)	8,992 (43.9)	24,649 (60.0)	0.31 (0.30–0.32)	5,890 (43.1)	3,102 (49.0)	0.88 (0.85–0.91)
65 +	12,941 (21.2)	34,290 (22.4)	8.651 (21.0)	0.38 (0.37–0.40)	3,162 (23.1)	1,128 (17.8)	0.99 (0.96–1.02)
Sex							
Male	37,253 (61.0)	12.366 (61.8)	24,887 (60.5)	Reference	8,747 (63.9)	3,619 (57.2)	Reference
Female	23,871 (39.0)	7,648 (38.2)	16,223 (39.5)	0.97 (0.94–0.99)	4,934 (36.1)	2,714 (428)	0.91 (0.89–0.93)
Cardiac Condition or Prosthetic Joint							
Cardiac Condition	15,707 (25.7)	14,107 (70.5)	1,600 (3.9)	Reference	9,586 (70.1)	4,521 (71.4)	Reference
Prosthetic Joint	38,445 (62.9)	0 (0.0)	38,445 (93.5)	–	–	–	–
Both	6,972 (11.4)	5,907 (29.5)	1,065 (2.6)	0.94 (0.93–0.95)	4,095 (29.9)	1,812 (28.6)	1.02 (0.99–1.04)
Region							
Northeast	7,901 (12.9)	2,915 (14.6)	4,986 (12.1)	Reference	1,864 (13.6)	1,051 (16.6)	Reference
Midwest	22,780 (37.3)	6,893 (34.4)	15,887 (38.7)	0.82 (0.79–0.85)	2,998 (36.5)	1,895 (29.9)	1.13 (1.10–1.17)
South	22,635 (37.0)	7,766 (38.8)	14,869 (36.2)	0.93 (0.90–0.96)	5,268 (38.5)	2,498 (39.4)	1.06 (1.03–1.09)
West	7,808 (12.8)	2,440 (12.2)	5,368 (13.0)	0.85 (0.81–0.88)	1,551 (11.3)	889 (14.0)	0.99 (0.95–1.04)
Location							
Rural	9,659 (15.8)	2,829 (14.1)	6,830 (16.6)	Reference	2,181 (15.9)	648 (10.2)	Reference
Urban	51,465 (84.2)	17,185 (85.9)	34,280 (83.4)	1.14 (1.10–1.18)	11,500 (84.1)	5,685 (89.8)	0.87 (0.85–0.89)
Year							
2015	20,684 (33.8)	6,684 (33.4)	14,000 (34.1)	Reference	4,594 (33.6)	2,090 (33.0)	Reference
2016	20,745 (22.9)	6,803 (34.0)	13,942 (33.9)	1.01 (0.99–1.04)	2,641 (33.9)	2,162 (34.1)	0.99 (0.97–1.12)
2017	19,695 (32.3)	6,527 (32.6)	13,168 (32.0)	1.03 (0.98–1.05)	4,446 (32.5)	2,081 (32.9)	0.99 (0.97–1.01)
*Comorbidities*							
Mean Charlson (SD)	0.91 (1.5)	1.2 (1.3)	0.75 (1.8)	1.21 (1.20–1.22)	1.23 (1.8)	1.22 (1.8)	1.00 (0.99–1.01)
Myocardial Infarction							
No	60,217 (98.5)	19,437 (97.2)	40,780 (99.2)	Reference	13,300 (97.2)	6,137 (96.9)	Reference
Yes	907 (1.5)	577 (2.8)	330 (0.8)	1.97 (1.87–2.07)	381 (2.8)	196 (3.1)	0.96 (0.91–1.02)
Congestive Heart Failure							
No	56,315 (92.1)	16,633 (83.1)	39,682 (96.5)	Reference	11,311 (82.7)	5,322 (84.0)	Reference
Yes	4,809 (7.9)	3,381 (16.9)	1,428 (3.5)	2.38 (2.33–2.43)	2,370 (17.3)	1,011 (16.0)	1.03 (1.01–1.06)
Peripheral Vascular Disease							
No	57,330 (93.8)	17,856 (89.2)	29,474 (96.0)	Reference	12,197 (89.2)	5,659 (89.4)	Reference
Yes	3,794 (6.2)	2,158 (10.8)	1,636 (4.0)	1.83 (1.77–1.88)	1,484 (10.8)	674 (10.6)	1.01 (0.98–1.04)
Cerebrovascular Disease							
No	58,824 (96.2)	18,611 (93.0)	40,213 (97.8)	Reference	12,725 (93.0)	5,886 (92.9)	Reference
Yes	2,300 (3.8)	1,403 (7.0)	897 (2.2)	1.93 (1.86–2.00)	956 (7.0)	447 (7.1)	00.99 (0.96–1.03)
Dementia							
No	60,811 (99.5)	19.852 (99.2)	40,959 (99.6)	Reference	13,558 (99.1)	6,294 (99.4)	Reference
Yes	313 (0.5)	162 (0.8)	151 (0.4)	1.59 (1.42–1.76)	123 (0.9)	39 (0.6)	1.11 (1.02–1.21)
COPD							
No	53,515 (87.6)	17,160 (85.7)	36,355 (88.4)	Reference	11,751 (85.9)	5,409 (85.4)	Reference
Yes	7,609 (12.4)	2,854 (14.3)	4,755 (11.6)	1.17 (1.13–1.21)	1,930 (14.1)	924 (14.6)	0.99 (0.96–1.01)
Connective Tissue Disease							
No	59,172 (96.8)	19,427 (97.1)	39,745 (96.7)	Reference	13,288 (97.1)	6,139 (96.9)	Reference
Yes	1,952 (3.2)	587 (2.9)	1,365 (3.3)	0.92 (0.86–0.98)	393 (2.9)	194 (3.1)	0.98 (0.92–1.04)
Peptic Ulcer Disease							
No	60,657 (99.2)	19,832 (99.1)	40,825 (99.3)	Reference	13,553 (99.1)	6,279 (99.2)	Reference
Yes	467 (0.8)	182 (0.9)	285 (0.7)	1.19 (1.06–1.34)	128 (0.9)	54 (0.8)	1.03 (0.94–1.13)
Liver Disease							
No	59,063 (96.6)	19,182 (95.8)	39,881 (97.0)	Reference	13.150 (96.1)	6,032 (95.3)	Reference
Mild	1,534 (2.5)	582 (2.9)	952 (2.3)	1.17 (1.09–1.25)	358 (2.6)	224 (3.5)	0.90 (0.84–0.96)
Moderate to Severe	527 (0.9)	250 (1.3)	277 (0.7)	1.46 (1.33–1.60)	173 (1.3)	77 (1.2)	1.91 (0.93–1.10)
Diabetes							
No	51.932 (85.0)	16,942 (84.6)	34,990 (85.1)	Reference	11,600 (84.8)	5,342 (84.4)	Reference
Uncomplicated	6,880 (11.3)	2,132 (10.7)	4,748 (11.6)	0.95 (0.92–0.99)	1,433 (10.5)	699 (11.0)	0.98 (0.95–1.02)
Complicated	2,312 (3.7)	940 (4.7)	1,372 (3.3)	1.25 (1.18–1.31)	648 (4.7)	292 (4.6)	1.01 (0.96–1.05)
Paraplegia/Hemiplegia							
No	60,826 (99.5)	19,857 (99.2)	40,969 (99.7)	Reference	113,575 (99.2)	6,282 (99.2)	Reference
Yes	298 (0.5)	157 (0.8)	141 (0.3)	1.61 (1.45–1.80)	106 (0.8)	51 (0.8)	0.99 (0.89–1.10)
Renal Disease							
No	56,615 (92.6)	17,850 (89.2)	38,765 (94.3)	Reference	12,161 (88.9)	5,689 (89.8)	Reference
Yes	4,509 (7.4)	2,164 (10.8)	2,345 (5.7)	1.52 (1.47–1.57)	1,520 (11.1)	6444 (10.2)	1.03 (1.00–1.06)
Cancer							
No	57,490 (94.1)	18,752 (93.7)	38,738 (94.2)	Reference	12,818 (93.7)	5,934 (93.7)	Reference
Yes	3,634 (5.9)	1,262 (6.3)	2,372 (5.8)	1.06 (1.02–1.11)	863 (6.3)	399 (6.3)	1.00 (0.96–1.04)
Metastatic Solid Tumor							
No	60,801 (99.5)	19.889 (99.4)	40,912 (99.5)	Reference	13,595 (99.4)	6,294 (99.4)	Reference
Yes	323 (0.5)	125 (0.6)	198 (0.5)	1.18 (1.03–1.36)	86 (0.6)	39 (0.6)	1.01 (0.89–1.13)
AIDS							
No	61,035 (99.9)	19,977 (99.8)	41,058 (99.9)	Reference	13,656 (99.8)	6,321 (99.8)	Reference
Yes	89 (0.1)	37 (0.2)	52 (0.1)	1.27 (0.99–1.63)	25 (0.2)	12 (0.2)	0.99 (0.79–1.24)
*Dental Procedures*							
Gingival Manipulation							
No	7,505 (12.3)	0 (0.0)	7,505 (18.3)	Reference	–-^b^	–-	–-
Yes	53,619 (87.7)	20,014 (100.0)	33,605 (81.7)	–-	–-	–-	–-
Adjunctive							
No	57,368 (93.9)	19,063 (95.2)	38.305 (93.2)	Reference	13,051 (95.4)	6,012 (94.9)	Reference
Yes	2,756 (6.1)	951 (4.8)	2,805 (6.8)	0.76 (0.72–0.81)	630 (4.6)	321 (5.1)	0.97 (0.92–1.01)
Diagnostic							
No	17,585 (28.8)	4,360 (21.8)	13.225 (32.2)	Reference	2,967 (21.7)	1,393 (22.0)	Reference
Yes	43,539 (72.2)	15,654 (78.2)	27.,885 (67.8)	1.45 (1.41–1.49)	10.714 (78.3)	4,940 (78.0)	1.01 (0.98–1.03)
Endodontics							
No	59,690 (97.7)	19.530 (97.6)	0.410	Reference	13,367 (97.7)	6,163 (97.3)	Reference
Yes	1,434 (2.3)	484 (2.4)	950 (2.3)	1.04 (0.96–1.11)	314 (2.3)	170 (2.7)	0.95 (0.89–1.01)
Implant							
No	60,268 (98.6)	19.756 (98.8)	40,512 (98.5)	Reference	13,520 (98.8)	6,236 (98.5)	Reference
Yes	856 (1.4)	258 (1.3)	598 (1.5)	0.92 (0.83–1.02)	161 (1.2)	97 (1.5)	0.91 (0.83–1.00)
Maxillofacial Prosthetics							
No	61,082 (99.9)	20,005 (99.9)	41,077 (99.9)	Reference	13.675 (99.9)	6,330 (99.9)	Reference
Yes	42 (0.1)	9 (0.1)	33 (0.1)	0.65 (0.37–1.17)	6 (0.1)	3 (0.1)	0.98 (0.61–1.55)
Oral Maxillofacial Surgery							
No	57,435 (94.0)	18,545 (92.7)	28,890 (94.6)	Reference	12,703 (92.8)	5,842 (92.3)	Reference
Yes	3,689 (6.0)	1,469 (7.3)	2,220 (5.4)	1.23 (1.18–1.29)	978 (7.2)	491 (7.8)	0.97 (0.94–1.01)
Orthodontics							
No	61,054 (99.9)	19,999 (99.9)	41,055 (99.9)	Reference	13,670 (99.9)	6,329 (99.9)	Reference
Yes	70 (0.1)	15 (0.1)	55 (0.1)	0.65 (0.42–1.02)	11 (0.1)	4 (0.1)	1.08 (0.79–1.46)
Periodontics							
No	56,376 (92.2)	18,275 (91.3)	38,101 (92.7)	Reference	12,535 (91.6)	5,740 (90.6)	Reference
Yes	4,748 (7.8)	1,739 (8.7)	3,009 (7.3)	1.13 (1.09–1.18)	1,146 (8.4)	593 (9.4)	0.96 (0.93–1.00)
Preventive							
No	31,436 (51.4)	9,192 (45.9)	22,244 (54.1)	Reference	6,334 (46.3)	2,858 (45.1)	Reference
Yes	29,688 (48.6)	10,822 (54.1)	18,866 (45.9)	1.25 (1.22–1.27)	7,347 (53.7)	2,475 (55.9)	0.99 (0.97–1.00)
Prosthodontics							
No	59,060 (96.6)	19,594 (97.9)	39,466 (96.0)	Reference	13,399 (97.9)	6,195 (97.8)	Reference
Yes	2,064 (3.4)	420 (2.1)	1,644 (4.0)	0.61 (0.56–0.67)	282 (2.1)	138 (2.2)	0.98 (0.91–1.05)
Fixed Prosthodontics							
No	60,327 (98.7)	19,920 (99.5)	40.407 (98.3)	Reference	13,617 (99.5)	6,303 (99.5)	Reference
Yes	797 (1.3)	94 (0.5)	703 (1.7)	0.36 (0.29–0.43)	64 (0.5)	30 (0.5)	1.00 (0.87–1.14)
Restorative							
No	46,350 (75.8)	16,184 (80.7)	30,202 (73.5)	Reference	11,047 (80.8)	5,101 (80.6)	Reference
Yes	14,774 (24.2)	3.866 (19.3)	10,908 (26.5)	0.75 (0.73–0.77)	2,634 (19.2)	1,232 (19.4)	1.00 (0.97–1.02)
Uncategorized							
No	60,717 (99.3)	19,913 (99.5)	40,804 (99.3)	Reference	13,607 (99.5)	6,306 (99.6)	Reference
Yes	407 (0.7)	101 (0.5)	306 (0.7)	0.76 (0.64–0.90)	74 (0.5)	27 (0.4)	1.07 (0.95–1.21)

^a^Concordance was based on medical condition (cardiac condition and/or prosthetic joint) and receipt of a procedure involving gingival manipulation and antibiotic agent; dose was not taken into consideration for this table

^b^By definition, all observations included for the sub-analysis were procedures involving gingival manipulation

Concordant prescribing differed by age group, sex, cardiac condition and/or prosthetic joint, region, and treatment setting (Table 1). Concordant prescribing also differed by Charlson score with those that had guideline concordant prescribing having a higher Charlson score than those with discordant prescribing. This relationship was also evident in most individual comorbidities. Those with preventive procedures (vs. those without) were more likely to have guideline concordant prescribing (unadjusted prevalence ratio (uPR) = 1.25, 95% CI: 1.22–1.27). A slightly lower percentage of those in the VA setting received a guideline concordant antibiotic (30.9%) than those in the non-VA setting (33.5%) (uPR = 0.92, 95% CI: 0.90–0.95) (Table 1).

### Multivariable regression for main analysis

Multivariable regression results can be found in Table 2. The model shows, after adjustment with age group, sex, prosthetic joint, region, treatment setting and dental procedures, that the prevalence of concordant prescribing was higher in the VA compared to non-VA dental settings (adjusted prevalence ratio (aPR)) = 1.21, 95% CI: 1.16–1.25). The reverse in association seen between the unadjusted and the multivariable regression results prompted further analysis to assess drivers of this association, where the addition of prosthetic joint variable increased the prevalence ratio the most. Therefore, this variable was assessed for interaction by stratifying the main prevalence ratio by those with and without prosthetic joints. The stratified results showed that among those without a prosthetic joint, the prevalence of guideline concordant prescribing was lower in the VA compared to non-VA settings (aPR = 0.82, 95% CI: 0.81–0.84). While among those with prosthetic joints, the prevalence of guideline concordant prescribing is higher in the VA setting (aPR = 1.73, 95% CI: 1.65–1.81). However, in multivariable results (Table 3), there was no longer a statistically significant difference in concordance between VA and non-VA settings in those without prosthetic joint (aPR = 0.99, 95% CI: 0.96–1.01). Among those with prosthetic joints, the prevalence of guideline concordant prescribing was still significantly higher in VA settings compared to non-VA settings in adjusted models (aPR = 1.73, 95% CI: 1.59–1.88).

**Table 2:** Multivariable model for overall concordant prescribing

Variables	Prevalence Ratio (95% CI)
Dental Setting (VA vs. Non-VA)	1.21 (1.16–1.25)
Age Group	
35–44 vs. 18–24	0.95 (0.93–0.97)
45–54 vs. 18–24	0.87 (0.85–0.88)
55–64 vs. 18–24	0.85 (0.84–0.87)
65 + vs. 18–24	0.97 (0.94–1.02)
Sex (Female vs. Male)	1.02 (1.00–1.03)
Prosthetic Joint (Yes vs. No)	0.15 (0.15–0.16)
Region	
Midwest vs. Northeast	0.96 (0.94–0.99)
South vs. Northeast	0.99 (0.96–1.01)
West vs. Northeast	0.92 (0.89–0.94)
Location (Urban vs. Rural)	1.02 (0.99–1.05)
Charlson	1.05 (1.05–1.06)
Adjunctive (Yes vs. No)	0.80 (0.77–0.84)
Diagnostic (Yes vs. No)	1.32 (1.29–1.35)
Periodontics (Yes vs. No)	1.36 (1.23–1.30)
Preventive (Yes vs. No)	1.14 (1.13–1.17)
Prosthodontics (Yes vs. No)	0.70 (0.64–0.75)
Fixed Prosthodontics (Yes vs. No)	0.46 (0.39–0.55)

^a^All covariates included in the table were used for adjustment in the regression analysis

**Table 3:** Multivariable model for overall concordant prescribing stratified by presence of a prosthetic joint

	Without Prosthetic Joint	With Prosthetic Joint
Variables	*N* = 15,707	*N* = 45,417
Dental Setting (VA vs. Non-VA)	0.99 (0.96–1.01)	1.73 (1.59–1.88)
Age Group		
35–44 vs. 18–24	0.98 (0.96–0.99)	0.68 (0.50–0.93)
45–54 vs. 18–24	0.98(0.96–0.99)	0.54 (0.41–0.70)
55–64 vs. 18–24	0.96 (0.95–0.97)	0.61 (0.47–0.80)
65 + vs. 18–24	0.94 (0.92–0.97)	0.87 (0.66–1.14)
Sex (Female vs. Male)	1.00 (0.99–1.01)	1.08 (1.01–1.14)
Region		
Midwest vs. Northeast	0.99 (0.9801.01)	0.92 (0.85–0.99)
South vs. Northeast	1.01 (0.99–1.02)	0.93 (0.87–1.00)
West vs. Northeast	1.02 (1.01–1.04)	0.74 (0.67–0.82)
Location (Urban vs. Rural)	0.99 (0.97–0.99)	1.07 (1.00–1.14)
Charlson	1.00 (0.99–1.00)	1.19 (1.17–1.20)
Adjunctive (Yes vs. No)	0.80 (0.77–0.84)	0.78 (0.70–0.87)
Diagnostic (Yes vs. No)	1.26 (1.24–1.28)	1.48 (1.40–1.57)
Periodontics (Yes vs. No)	1.23 (1.21–1.25)	1.34 (1.22–1.47)
Preventive (Yes vs. No)	1.12 (1.11–1.14)	1.16 (1.09–1.23)
Prosthodontics (Yes vs. No)	0.65 (0.60–0.71)	0.74 (0.65–0.84)
Fixed Prosthodontics (Yes vs. No)	0.50 (0.41–0.61)	0.41 (0.30–0.59)

### Sub-analysis of guideline concordance of dosing

Of the 20,014 concordant prescriptions, 6,333 (31.6%) had concordant dosing based on duration of the antibiotic. Concordant dosing occurred in 80.5% of VA prescriptions compared to 66.2% in the non-VA setting. After adjustment in multivariable analyses, the prevalence of concordant dosing in VA was higher than in non-VA dental settings (aPR = 1.11, 95% CI: 1.07–1.15; Table 4).

**Table 4:** Results of Multivariable Log Binomial Regression: Factors associated with guideline concordant dosing based on antibiotic duration

Variables	Prevalence Ratio (95% CI)
Dental Setting (VA vs. Non-VA)	1.11 (1.07–1.15)
Age Group	
35–44 vs. 18–24	0.89 (0.85–0.93)
45–54 vs. 18–24	0.89 (0.86–0.93)
55–64 vs. 18–24	0.86 (0.83–0.88)
65 + vs. 18–24	0.86 (0.82–0.91)
Sex (Female vs. Male)	0.94 (0.92–0.96)
Region	
Midwest vs. Northeast	1.12 (1.09–1.16)
South vs. Northeast	1.05 (1.02–1.08)
West vs. Northeast	0.98 (0.94–1.02)
Location (Urban vs. Rural)	0.88 (0.86–0.90)
Charlson	1.00 (0.99–1.01)
Implant (Yes vs. No)	0.90 (0.82–0.99)

## Discussion

This study is the first to compare guideline concordant antibiotic prophylaxis for dental treatment by VA and non-VA dental settings. This study found that among those with prosthetic joints, the prevalence of guideline concordant prescribing of antibiotic prophylaxis is 73% higher in VA settings whereas no significant difference was identified in in those without a prosthetic joint. Prevalence of guideline concordance was 11% greater in VA settings when assessing errors of antibiotic dosing based on antibiotic duration.

Other studies have assessed antibiotic prescriptions prior to dental treatment exclusively in only one setting, VA or non-VA. Though different studies have examined appropriate dental prescriptions within the VA setting, the procedures and inclusion/exclusion were different and make it difficult to compare to this study. One VA dental study found that 43% of discordant antibiotic prescriptions were prescribed for prophylaxis [29]. However, the study did not provide the proportion of discordant prescribing among all prophylaxis prescriptions, making it difficult to compare with our study. Another VA study utilizing 2015 data assessed appropriate antibiotic prophylaxis of veterans undergoing dental visits for tooth extractions, dental implants, and periodontal surgical procedures using the AHA, AAOS, ADA guidelines, and systematic Cochrane reviews [30]. The study found that over three-fourths (87.3%) of those who should have received prophylaxis actually received an antibiotic. However, 84.9% of those that should have received only one antibiotic dose pre-procedure also received an extended course of antibiotics post procedure. Our study focused on pre-procedural antibiotics and did not assess patients that did not receive an antibiotic when one was indicated. A national study by Durkin et al. focused on non-VA pharmacy benefits manager data found that of 6.2 million antibiotic prescriptions by general dentists between 2013–2015, 850,000 (13.7%) were prescribed inappropriately [31]. The disparity between our findings and Durkin et al. is due to the classification of inappropriate prescriptions. Durkin et al. did not consider any medical conditions or dental procedures and only focused on treatment duration and antibiotic agent. Another study, by Hubbard et al., utilizing the same Marketscan data as this study found unnecessary antibiotic prophylaxis ranged from 77–78.5% between 2016–2018 [32]. However, that study did not limit its analysis to those with a cardiac condition or prosthetic joint, which is why the proportion of inappropriate antibiotic prescriptions identified is higher than in our study.

One unique finding in this study, was the differing results by dental setting when stratifying the assessment of guideline concordance by those with and without prosthetic joints. A study by Suda et al. found that presence of a prosthetic joint was associated with unnecessary prophylactic prescriptions, with 88.7% prescribed an unnecessary antibiotic prophylaxis [8]. Despite the adjusted findings, the VA also had a higher proportion (81.7%) of those with a prosthetic joint receiving an unnecessary antibiotic, suggesting that both dental settings should work on improving discordant prescribing among this population. Additionally, further investigation into why guideline concordance differs among non-VA and VA settings among those with prosthetic joints exists is important to improving prescribing in this group for both settings. Improving antibiotic prescribing is particularly important as use of antibiotics can led to serious adverse events, including *C. difficile* and multi-drug resistant organisms [33–36].

Multiple factors may be driving differences in prescribing practices seen in this study between the VA and non-VA settings. Results of this study suggest that some aspect or factor of the VA may be leading to better guideline concordant antibiotic prophylaxis. It is possible, that VA dentists, with access to an integrated EHR, are better able to synthesize medical information to guide their decision making. However, there are a number of factors that can lead to concordant prescribing including provider level, patient level and, system level factors [15, 16]. This study did not have access and did not include system level factors, provider factors, and many patient factors (i.e., patient demands or request, physician expectation for prophylaxis) that could have influenced these results. Therefore, future studies should focus on how integrated EHR use makes a direct impact in antibiotic prophylaxis and what other factors may be influencing prescribing.

### Limitations & strengths

This study has limitations. First, it could not account for all population or systemic differences (e.g., antibiotic stewardship programs, formulary restrictions) between VA and non-VA settings. Second, this study was not able to capture actual use of the integrated EHR to guide prescribing within the analysis and that the presence of an integrated EHR in non-VA settings is unknown. A third limitation was the inability to identify the provider type prescribing the prophylactic antibiotic in non-VA data. To address this issue, analogous approaches were used to identify each cohort to ensure that each dataset use similar methods to identify dental prescribing. Nevertheless, this method does not capture differences in provider knowledge and experience that may be causing variation in prescribing practices. Fourth, these analyses focused on a commercially insured non-veteran patient population and a veteran population with poorer oral health and more comorbidities [37]. As such, the results of this study are not generalizable beyond these cohorts. Fifth, the VA cohort was predominantly composed of men, and results may not be generalizable to women. Sixth, there was the potential for misclassification of appropriate prophylaxis indication. This study used the assumption that antibiotics included were for dental prophylaxis based on the temporal relationship of the prescription to the dental encounter, consistent with previously applied criteria. Seventh, we used ICD-9 -CM and ICD-10 -CM codes to identify the patient population. If patients with a cardiac condition or prosthetic joint were miscoded, they would not be included within this analysis resulting in selection bias. Furthermore, there is the potential for misclassification bias for consecutive dental appointment beyond 7 days, or if combined appointments within 7-day windows were not related. Finally, we did not assess guideline concordance according to expert opinion presented in the AAOS Appropriate Use Criteria (AUC) for Management of Patients with Orthopaedic Implants Undergoing Dental Procedures [38]. However, based on our prior work, application of the AUC would not significantly alter our results [8, 39].

Despite these limitations, analytic cohorts were defined with specific and reproducible criteria aimed to identify comparable patient populations. This study advances the knowledge of the topic by evaluating national data to compare VA and non-VA dental settings. This study also used two of the largest and most comprehensive datasets for dental research available. Furthermore, a strength of this study includes a first step on understanding how antibiotic prophylaxis differs across dental settings, which has not been studied previously.

## Conclusions

The prevalence of guideline concordant prescribing for antibiotic prophylaxis among those with cardiac conditions and prosthetic joints is higher in the VA compared to non-VA setting. Guideline concordant prescribing was found to be 73% greater in the VA dental setting vs. the non-VA setting in patients with prosthetic joints but not statistically different in those without a prosthetic joint. Guideline concordant dosing was also 11% greater in the VA vs. non-VA settings in patients who received a guideline concordant antibiotic prescription. There is a possibility that system level factors such as availability of an integrated EHR, or other prescriber related factors impacted findings. Future studies should focus on understanding specific system and provider level factors that may improve guideline concordant prescribing.

## Supplementary Information

**Additional file 1: Supplemental Table 1.** Distribution of characteristics by dental setting.

## Data Availability

We are committed to collaborating and sharing these data to maximize their value to improve veterans and others’ health and health care, to the greatest degree consistent with current Veterans Affairs regulations and policy. We can provide access to the programming code used to identify the sample and conduct analyses and will conduct additional analyses as requested. We cannot provide a link to the database directly as it would compromise patients’ anonymity, and permissions from VA are needed to obtain the data. The Marketscan dataset used for this study is proprietary and unable to be shared. Interested individuals may contact the corresponding author (Dr. KJ Suda) for information.
