# Supplementary material for: Comparison of guideline concordant antibiotic prophylaxis in Veterans Affairs and non-Veterans Affairs dental settings among those with cardiac conditions or prosthetic joints
Source: BMC Infect Dis. 2023 Jun 23;23:427. doi: 10.1186/s12879-023-08400-y (PMC10290301; doi:10.1186/s12879-023-08400-y)
Supplement: Supplementary file 1 — Additional file 1: Supplemental Table 1. Distribution of characteristics by dental setting. [file 12879_2023_8400_MOESM1_ESM.docx]

**Appendix. Supplemental Table**

Supplemental Table 1. Distribution of characteristics by dental setting

| Variables | Total  N=61,124 | VA (%)  N=18,292 | Non-VA (%)  N=42,832 | Unadjusted Prevalence Ratio  (95% CI) |
| --- | --- | --- | --- | --- |
| *Demographics and Clinical Characteristics* | | | | |
| Mean Age (SD) | 59.1 (10.4); [18,99] | 67.1 (9.5) | 55.7 (8.9) | 1.08 (1.07-1.08) |
| Age Group |  |  |  |  |
| 18-24 | 1,799 (2.9) | 45 (0.3) | 1,754 (4.1) | Reference |
| 35-44 | 2,361 (3.9) | 224 (1.2) | 2,137 (5.0) | 3.79 (2.77-5.19) |
| 45-54 | 10,382 (17.0) | 1,304 (7.1) | 9.078 (21.2) | 5.02 (2.75-6.73) |
| 55-64 | 33,641 (55.0) | 4,077 (22.3) | 29,564 (69.0) | 4.84 (3.63-6.47) |
| 65+ | 12,941 (21.2) | 12,642 (69.1) | 299 (0.7) | 39.05 (29.26-52.1) |
| Sex |  |  |  |  |
| Male | 37,253 (61.0) | 17,248 (94.3) | 20,005 (46.7) | Reference |
| Female | 23,871 (39.0) | 1,044 (5.7) | 22,827 (53.3) | 0.09 (0.08-0.10) |
| Cardiac condition |  |  |  |  |
| No | 38,445 (62.9) | 10,945 (59.8) | 27,500 (64.2) | Reference |
| Yes | 22,679 (37.1) | 7,347 (40.2) | 15,332 (35.8) | 1.14 (1.11-1.17) |
| Prosthetic Joint |  |  |  |  |
| No | 15,707 (25.7) | 3,938 (21.5) | 11,769 (27.5) | Reference |
| Yes | 45,427 (74.3) | 14,354 (78.5) | 31,063 (72.5) | 1.26 (1.22-1.30) |
| Cardiac Condition or Prosthetic Joint |  |  |  |  |
| Cardiac Condition | 15,707 (25.7) | 3,938 (21.5) | 11,769 (27.5) | Reference |
| Prosthetic Joint | 38,445 (62.9) | 10,945 (59.8) | 27,500 (64.2) | 1.14 (1.10-1.17) |
| Both | 6,972 (11.4) | 3,409 (18.6) | 3,563 (8.3) | 1.95 (1.88-2.02) |
| Region |  |  |  |  |
| Northeast | 7,901 (12.9) | 2,284 (12.5) | 5,617 (13.1) | Reference |
| Midwest | 22,780 (37.3) | 5,102 (27.9) | 17,678 (41.3) | 0.77 (0.74-0.81) |
| South | 22,635 (37.0) | 8.421 (46.0) | 14,214 (33.2) | 1.29 (1.24-1.34) |
| West | 7,808 (12.8) | 2,485 (13.6) | 5,323 (12.4) | 1.10 (1.05-1.15) |
| Location |  |  |  |  |
| Rural | 9,659 (15.8) | 3,014 (16.5) | 6,645 (15.5) | Reference |
| Urban | 51,465 (84.2) | 15,278 (83.5) | 26,187 (84.5) | 0.95 (0.92-0.98) |
| Year |  |  |  |  |
| 2015 | 20,684 (33.8) | 6,051 (33.1) | 14,633 (34.2) | Reference |
| 2016 | 20,745 (22.9) | 6,210 (34.0) | 14,535 (33.9) | 1.02 (0.99-1.05) |
| 2017 | 19,695 (32.3) | 6,031 (32.9) | 13,664 (31.9) | 1.05 (1.02-1.08) |
| Guideline Concordant |  |  |  |  |
| No | 41,110 (67.3) | 12,629 (69.0) | 28,481 (66.5) | Reference |
| Yes | 20,014 (32.7) | 5,663 (31.0) | 14,351 (33.5) | 0.92 (0.89-0.95) |
| *Comorbidities* | | | | |
| Mean Charlson (SD) | 0.91 (1.5) [0,17] | 0.8 (1.7) | 1.0 (1.5) | 0.92 (0.91-0.0.94) |
| Myocardial Infarction |  |  |  |  |
| No | 60,217 (98.5) | 17.73 (98.3) | 42,244 (98.6) | Reference |
| Yes | 907 (1.5) | 319 (1.7) | 588 (1.4) | 1.18 ((1.08-1.29) |
| Congestive Heart Failure |  |  |  |  |
| No | 56,315 (92.1) | 17,098 (93.5) | 39,217 (91.6) | Reference |
| Yes | 4,809 (7.9) | 1,194 (6.5) | 3,615 (8.4) | 0.82 (0.78-0.86) |
| Peripheral Vascular Disease |  |  |  |  |
| No | 57,330 (93.8) | 17,840 (97.5) | 39,490 (92.2) | Reference |
| Yes | 3,794 (6.2) | 452 (2.5) | 3,342 (7.8) | 0.38 (0.35-0.42) |
| Cerebrovascular Disease |  |  |  |  |
| No | 58,824 (96.2) | 17,866 (97.7) | 40,958 (95.6) | Reference |
| Yes | 2,300 (3.8) | 426 (2.3) | 1,874 (4.4) | 0.61 (0.56-0.66) |
| Dementia |  |  |  |  |
| No | 60,811 (99.5) | 18,090 (98.9) | 42,721 (99.7) | Reference |
| Yes | 313 (0.5) | 202 (1.1) | 111 (0.3) | 2.17 (2.00-2.36) |
| COPD |  |  |  |  |
| No | 53,515 (87.6) | 16,820 (92.0) | 36,695 (85.6) | Reference |
| Yes | 7,609 (12.4) | 1,472 (8.0) | 6,137 (14.3) | 0.62 (0.59-0.66) |
| Connective Tissue Disease |  |  |  |  |
| No | 59,172 (96.8) | 18.136 (99.2) | 41,036 (95.8) | Reference |
| Yes | 1,952 (3.2) | 156 (0.8) | 1,796 (4.2) | 0.26 (0.22-0.31) |
| Peptic Ulcer Disease |  |  |  |  |
| No | 60,657 (99.2) | 18.185 (99.4) | 42,472 (99.2) | Reference |
| Yes | 467 (0.8) | 107 (0.6) | 360 (0.8) | 0.76 (0.65-0.90) |
| Liver Disease |  |  |  |  |
| No | 59,063 (96.6) | 17,893 (97.8) | 41,170 (96,1) | Reference |
| Mild | 1,534 (2.5) | 219 (1.2) | 1,315 (3.1) | 0.47 (0.42-0.53) |
| Moderate to Severe | 527 (0.9) | 180 (1.0) | 347 (0.8) | 1.13 (1.00-1.27) |
| Diabetes |  |  |  |  |
| No | 51.932 (85.0) | 15,983 (87.4) | 35,949 (83.9) | Reference |
| Uncomplicated | 6,880 (11.3) | 1,417 (7.8) | 5,463 (12.8) | 0.67 (0.62-0.70) |
| Complicated | 2,312 (3.7) | 892 (4.8) | 1,420 (3.3) | 1.25 (1.19-1.32) |
| Paraplegia/Hemiplegia |  |  |  |  |
| No | 60,826 (99.5) | 18,195 (99.5) | 42,631 (99.5) | Reference |
| Yes | 298 (0.5) | 97 (0.5) | 201 (0.5) | 1.09 (0.92-1.28) |
| Renal Disease |  |  |  |  |
| No | 56,615 (92.6) | 16,580 (90.6) | 40,035 (93.5) | Reference |
| Yes | 4,509 (7.4) | 1,712 (9.4) | 2,797 (6.5) | 1.29 (1.25-1.35) |
| Cancer |  |  |  |  |
| No | 57,490 (94.1) | 17,612 (96.3) | 39,878 (93.1) | Reference |
| Yes | 3,634 (5.9) | 680 (3.7) | 2,954 (6.9) | 0.61 (0.57-0.65) |
| Metastatic Tumor |  |  |  |  |
| No | 60,801 (99.5) | 18,232 (99.7) | 42,569 (99.4) | Reference |
| Yes | 323 (0.5) | 60 (0.3) | 263 (0.6) | 0.62 (0.49-0.79) |
| AIDS |  |  |  |  |
| No | 61,035 (99.9) | 18,260 (99.8) | 42,775 (99.9) | Reference |
| Yes | 89 (0.1) | 32 (0.2) | 57 (0.1) | 1.20 (0.91-1.59) |
| *Dental Procedures* | | | | |
| Gingival Manipulation |  |  |  |  |
| No | 7,505 (12.3) | 4,304 (23.5) | 3,201 (7.5) | Reference |
| Yes | 53,619 (87.7) | 13,988 (76.5) | 36,631 (92.5) | 0.45 (0.44-0.47) |
| Adjunctive |  |  |  |  |
| No | 57,368 (93.9) | 16,034 (87.7) | 41,334 (96.5) | Reference |
| Yes | 2,756 (6.1) | 2,258 (12.3) | 1,498 (3.5) | 2.15 (2.09-2.21) |
| Diagnostic |  |  |  |  |
| No | 17,585 (28.8) | 7,129 (39.0) | 10,456 (24.4) | Reference |
| Yes | 43,539 (72.2) | 11,163 (61.0) | 32,375 (75.6) | 0.63 (0.62-0.65) |
| Endodontics |  |  |  |  |
| No | 59,690 (97.7) | 17,839 (97.5) | 41,851 (97.7) | Reference |
| Yes | 1,434 (2.3) | 453 (2.5) | 981 (2.3) | 1.06 (0.97-1.14) |
| Implant |  |  |  |  |
| No | 60,268 (98.6) | 17,839 (97.5) | 42,429 (99.1) | Reference |
| Yes | 856 (1.4) | 453 (2.5) | 403 (0.9) | 1.79 (1.68-1.91) |
| Maxillofacial Prosthetics |  |  |  |  |
| No | 61,082 (99.9) | 18,256 (99.8) | 42,826 (99.9) | Reference |
| Yes | 42 (0.1) | 36 (0.2) | 6 (0.1) | 2.87 (2.53-3.24) |
| Oral Maxillofacial Surgery |  |  |  |  |
| No | 57,435 (94.0) | 16,287 (89.0) | 41,148 (96.1) | Reference |
| Yes | 3,689 (6.0) | 2,005 (11.0) | 1,684 (3.9) | 1.92 (1.86-1.98) |
| Orthodontics |  |  |  |  |
| No | 61,054 (99.9) | 18,285 (99.9) | 42,769 (99.9) | Reference |
| Yes | 70 (0.1) | 7 (0.1) | 63 (0.1 | 0.33 (0.17-0.67) |
| Periodontics |  |  |  |  |
| No | 56,376 (92.2) | 17,597 (96.2) | 38,779 (90.5) | Reference |
| Yes | 4,748 (7.8) | 695 (3.8) | 4,053 (9.5) | 0.47 (0.44-0.50) |
| Preventive |  |  |  |  |
| No | 31,436 (51.4) | 15,774 (86.2) | 15,662 (36.6) | Reference |
| Yes | 29,688 (48.6) | 2,518 (13.8) | 27.170 (63.4) | 0.17 (0.16-0.18) |
| Prosthodontics |  |  |  |  |
| No | 59,060 (96.6) | 16,427 (89.8) | 42,633 (99.5) | Reference |
| Yes | 2,064 (3.4) | 1,865 (10.2) | 199 (0.5) | 3.25 (3.19-3.31) |
| Fixed Prosthodontics |  |  |  |  |
| No | 60,327 (98.7) | 17,697 (96.8) | 42,630 (99.5) | Reference |
| Yes | 797 (1.3) | 595 (3.3) | 202 (0.5) | 2.54 (2.44-2.65) |
| Restorative |  |  |  |  |
| No | 46,350 (75.8) | 13,061 (71.4) | 33,289 (77.7) | Reference |
| Yes | 14,774 (24.2) | 5,231 (28.6) | 9.543 (22.3) | 1.26 (1.22-1.30) |
| Uncategorized |  |  |  |  |
| No | 60,717 (99.3) | 17,937 (98.1) | 42,780 (99.9) | Reference |
| Yes | 407 (0.7) | 355 (1.9) | 52 (0.1) | 2.95 (2.84-3.07) |
